# A pilot investigation of genetic and epigenetic variation of FKBP5 and response to exercise intervention in African women with obesity

**DOI:** 10.1038/s41598-022-15678-6

**Published:** 2022-07-11

**Authors:** Tarryn Willmer, Amberly Oosthuizen, Stephanie Dias, Amy E. Mendham, Julia H. Goedecke, Carmen Pheiffer

**Affiliations:** 1 Biomedical Research and Innovation Platform, South African Medical Research Council, Tygerberg, 7505 South Africa; 2 Division of Medical Physiology, Centre for Cardiometabolic Research in Africa (CARMA), Faculty of Medicine and Health Sciences, University of Stellenbosch, Tygerberg, 7505 South Africa; 3 South African Medical Research Council/WITS Developmental Pathways for Health Research Unit (DPHRU), Department of Paediatrics, School of Clinical Medicine, Faculty of Health Sciences, University of the Witwatersrand, Johannesburg, South Africa; 4 Division of Physiological Sciences, Health Through Physical Activity, Lifestyle and Sport Research Centre (HPALS), Department of Human Biology, Faculty of Health Sciences, FIMS International Collaborating Centre of Sports Medicine, University of Cape Town, Cape Town, South Africa; 5 Non-Communicable Diseases Research Unit, South African Medical Research Council, Tygerberg, 7505, Cape Town, South Africa; 6 Department of Obstetrics and Gynaecology, Faculty of Health Sciences, University of Pretoria, Pretoria, 0001 South Africa

**Keywords:** Cell biology, Genetics, Molecular biology

## Abstract

We investigated gluteal (GSAT) and abdominal subcutaneous adipose tissue (ASAT) DNA methylation of *FKBP5* in response to a 12-week intervention in African women with obesity, as well as the effect of the rs1360780 single nucleotide polymorphism (SNP) on *FKBP5* methylation, gene expression and post-exercise training adaptations in obesity and metabolic related parameters. Exercise (n = 19) participants underwent 12-weeks of supervised aerobic and resistance training while controls (n = 12) continued their usual behaviours. *FKBP5* methylation was measured in GSAT and ASAT using pyrosequencing. SNP and gene expression analyses were conducted using quantitative real-time PCR. Exercise training induced *FKBP5* hypermethylation at two CpG dinucleotides within intron 7. When stratified based on the rs1360780 SNP, participants with the CT genotype displayed *FKBP5* hypermethylation in GSAT (*p* < 0.05), and ASAT displayed in both CC and CT carriers. CC allele carriers displayed improved cardiorespiratory fitness, insulin sensitivity, gynoid fat mass, and waist circumference (*p* < 0.05) in response to exercise training, and these parameters were attenuated in women with the CT genotype. These findings provide a basis for future studies in larger cohorts, which should assess whether *FKBP5* methylation and/or genetic variants such as the rs1360780 SNP could have a significant impact on responsiveness to exercise interventions.

## Introduction

The obesity pandemic has been largely driven by the consumption of unhealthy, western diets coupled with sedentary lifestyles^1^. These metabolic stressors have been hypothesized to contribute to obesity by disrupting the hypothalamic–pituitary–adrenal (HPA) axis, a neuro-endocrine pathway with essential roles in the stress response^2^, through a combination of genetic and epigenetic modifications in HPA-axis regulatory genes^3^.

FK506-binding protein (FKBP5) is a stress responsive co-chaperone of the glucocorticoid receptor (GR) and negative regulator of the HPA axis^4^. Its expression is rapidly induced by glucocorticoids, after which it attenuates GR signalling by restricting hormone binding and translocation of the GR complex to the nucleus, thus forming an ultra-short negative feedback loop^5^. Numerous genetic variants of *FKBP5* have been identified. Of these, the rs1360780 (C/T) single nucleotide polymorphism (SNP), located in intron 2, is the most extensively studied and has been demonstrated to alter *FKBP5* gene expression by modulating DNA methylation dynamics and transcription factor binding at multiple intronic enhancer regions within the *FKBP5* gene^6^.

While most FKBP5 research has examined its relationship with neuropsychiatric diseases, several recent studies have uncovered a role for this gene in metabolic control and obesity^7^. Preclinical studies in animal models have shown that gene deletion or pharmacological inhibition of FKBP5 leads to reduced white adipose tissue mass, and protection from diet-induced weight gain, insulin resistance and hepatic steatosis^8,9^. In humans, *FKBP5* expression in abdominal subcutaneous adipose tissue was shown to positively associate with markers of insulin resistance and type 2 diabetes^10^. Moreover, the *FKBP5* rs1360780 SNP has been associated with increased susceptibility to metabolic dysregulation, type 2 diabetes as well as decreased weight loss following bariatric surgery^11,12^. FKBP5 is also epigenetically regulated and two independent studies, conducted in peripheral blood and subcutaneous adipose tissue, reported an association between intronic *FKBP5* hypermethylation and several obesity and cardiometabolic traits^13,14^.

The excessive accumulation and dysfunction of white adipose tissue is considered a primary hallmark of obesity^15^. White adipose tissue is localized throughout the body in distinct depots which have been shown to vary in their association with metabolic diseases^16,17^. It is widely accepted that central adipose depots (including visceral and abdominal subcutaneous fat) are associated with greater risk of obesity-related complications, while lower-body fat (gluteo-femoral) is thought to be protective against metabolic disease^18^. Differences in these associations have however been reported in Black African and African American individuals^19,20^, thus highlighting the need for additional studies in these populations. Although the molecular basis for adipose depot-specific differences remain elusive, numerous studies have shown that abdominal and gluteal fat depots display distinct epigenetic and transcriptome profiles which may account for their functional differences and consequences for disease^21,22^.

Exercise training has been widely recognized as a favourable intervention to reduce the risks of developing obesity and metabolic disease^23,24^. Within adipose tissue, exercise training has been shown to reduce adipocyte size, modulate the expression of pro-inflammatory adipokines, decrease the induction of reactive oxygen species and inflammation, and increase vasculature; all processes that are dysregulated during obesity^16,25,26^. As a result, exercise has been consistently used as a model to assess the underlying mechanisms of insulin resistance and obesity^27^. To our knowledge, no studies have explored the genetic and epigenetic effects of exercise training on insulin resistance in Black African women with obesity. Accordingly, we firstly aimed to investigate abdominal and gluteal adipose tissue DNA methylation of *FKBP5* in response to a 12-week exercise intervention in Black African women with obesity. Secondly, we examined the depot-specific effect of the rs1360780 SNP on (1) *FKBP5* methylation, (2) *FKBP5* gene expression and in (3) response to the exercise intervention, as measured by obesity and metabolic parameters.

## Results

### Participant characteristics and response to the intervention

The characteristics of participants in response to the intervention have been previously described^27^ and are summarised in Table 1. In response to 12-weeks of exercise training there was a significant increase in peak oxygen consumption (VO_2peak_, *p* < 0.01 for interaction), an indicator of cardiorespiratory fitness, as well as insulin sensitivity (*p* = 0.049 for interaction). A modest but significant decrease in waist circumference (WC) was observed in the exercise group post intervention, whilst it increased in the control group (*p* < 0.001 for interaction). The control group also displayed increased body mass index (BMI, *p* = 0.006 for interaction) and weight (*p* = 0.006 for interaction) post intervention. Furthermore, exercise participants displayed a decrease in gynoid fat mass (FM, *p* = 0.003 for interaction) and an increase in circulating triglycerides levels (*p* = 0.001 for interaction) in response to the intervention.

**Table 1:** Participant characteristics pre- and post-intervention.

Variable	Control (n = 12)		Exercise (n = 19)		Group	Time	Interaction
	Pre	Post	Pre	Post	*P* value	*P* value	*P* value
Age (years)	24 (21–28)	–	22 (21–24)	–	–	–	–
VO_2peak_ (mL/min)	2085 ± 313	2022 ± 212	2090 ± 210	2289 ± 232*	0.961	0.406	**0.007**
VO_2max_ (mL/kg/min)	23.5 ± 3.0	22.6 ± 2.6	24.8 ± 2.4	27.4 ± 3.3*	0.211	0.286	**0.001**
**Body composition and fat distribution**							
Weight (kg)	83.6 ± 26.6	84.6 ± 20.5*	84.9 ± 8.2	84.1 ± 9.2	0.306	**0.047**	**0.006**
BMI (kg/m^2^)	33.0 (31.1–36.5)	33.3 (31.6–36.5) *	34.9 (32.8–36.4)	34.7 (30.4–37.0)	0.399	**0.047**	**0.006**
WHR	0.87 (0.1)	0.90 (0.1)	0.91 (0.1)	0.89 (0.1)	0.150	**0.034**	**0.005**
WC (cm)	97.0 ± 31.1	100.0 ± 24.8*	103.9 ± 7.4	100.7 ± 8.8*	0.886	**0.003**	** < 0.001**
VAT (cm^3^)	981.6 ± 450.6	1014.2 ± 407.6	931.4 ± 318.0	916.3 ± 343.9	0.662	0.276	0.244
Abdominal SAT (cm^3^)	5630.2 ± 1873.5	5662.2 ± 1968.2*	5603.9 ± 945.2	5567.0 ± 1169.3	0.915	**0.044**	**0.065**
FM (%)	49.1 (47.0–51.9)	49.6 (47.3–52.3)	50.5 (48.5–52.6)	50.2 (48.3–52.8)	0.305	0.695	0.579
Android FM (%)	8.3 ± 1.4	8.1 ± 1.6	8.3 ± 1.0	8.2 ± 1.0	0.901	0.098	0.781
Gynoid FM (%)	19.4 ± 2.6	19.5 ± 2.6	18.4 ± 1.7	18.2 ± 1.6*	0.217	0.197	**0.003**
**Glucose regulation**							
HbA1C (%)	5.3 ± 0.4	5.4 ± 0.3	5.2 ± 0.3	5.2 ± 0.3	0.327	0.365	0.653
HOMA-IR	2.9 (2.4–4.3)	3.4 (2.8–4.3)	3.6 (1.6–5.2)	3.2 (2.1–4.7)	0.856	0.663	0.543
Fasting Glucose (mmol/L)	4.9 ± 0.6	5.1 ± 0.8	5.5 ± 0.8	5.1 ± 1.0	**0.048**	0.533	0.148
Fasting Insulin (μU/ml)	15.4 ± 11.1	15.6 ± 10.2	14.6 ± 7.2	14.4 ± 6.3	0.428	0.853	0.926
S_I_ (× 10^-4^ min^−1^/(μU mL^−1^))	2.0 (1.3;3.2)	1.8 (1.6;2.6)	2.0 (1.2–2.8)	2.2 (1.5–3.7) *	0.204	0.680	**0.049**
**Lipid profile**							
Total cholesterol (mmol/L)	3.6 (3.0–4.4)	3.3 (2.7–4.1)	4.2 (3.3–4.4)	4.0 (3.7–5.1)	0.703	0.240	0.122
LDL (mmol/L)	2.0 (1.6–2.7)	1.7 (1.4–2.9)	2.7 (2.0–3.1)	2.7 (1.9–3.5)	0.301	0.388	0.442
HDL (mmol/L)	0.9 (0.8–1.1)	1.0 (0.8–1.1)	1.0 (0.8–1.2)	1.1 (0.9–1.2)	0.442	0.761	0.825
Triglycerides (mmol/L)	0.8 (0.6–1.1)	0.7 (0.5–0.9)	0.7 (0.6–0.8)	0.9 (0.7–1.0) *	0.092	0.187	**0.001**

Significant values are in bold.

Data presented as mean ± SD or as the median and interquartile range (25–75th percentile) for skewed variables.

*BMI* body mass index, *FM* fat mass, *HbA1C* haemoglobin A1C, *HDL* high-density lipoprotein, *HbA1C* haemoglobin A1C, *HOMA-IR* homeostatic model assessment of insulin resistance, *LDL* low-density lipoprotein, *SAT* subcutaneous adipose tissue, *Si* insulin sensitivity, *WHR* waist-to-hip ratio, *WC* waist circumference, *VAT* visceral adipose tissue.

*p < 0.05, significant within group change in response to the intervention.

### Impact of exercise intervention on FKBP5 methylation

**Figure 1:**
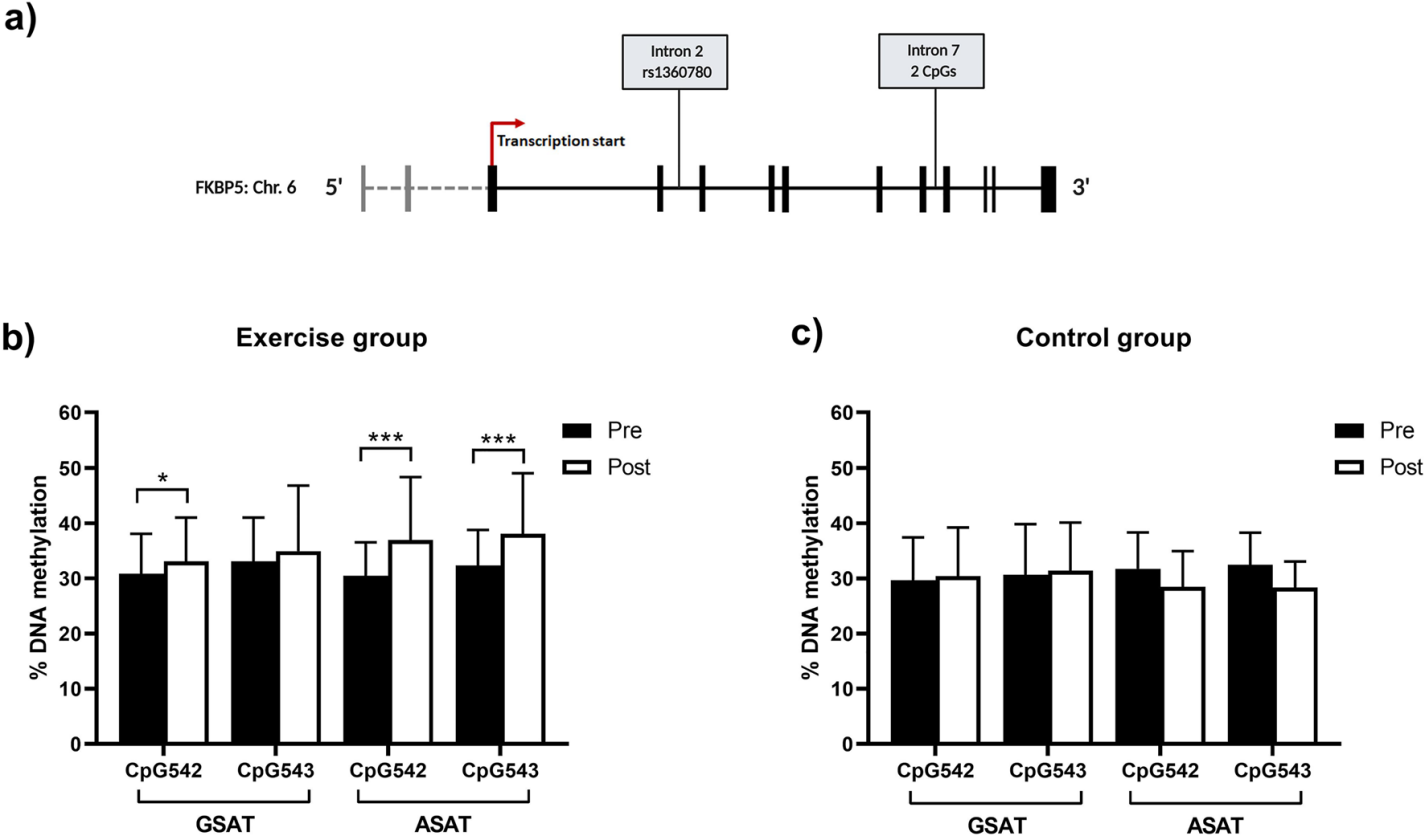
(**a**) Schematic diagram of the *FKBP5* gene, illustrating the rs1360780 SNP and CpG542/3 sites in introns 2 and 7, respectively. Pyrosequencing analysis of percentage methylation at *FKBP5* CpG542 and CpG543 in gluteal adipose tissue (GSAT) and abdominal adipose tissue (ASAT) in exercise (n = 19) (**b**) and control (n = 12) (**c**) participants pre- and post-intervention. Data represented as mean ± SD, *p < 0.05, ***p < 0.001.

We employed targeted bisulfite pyrosequencing to analyse the impact of exercise training on DNA methylation of two CpG sites situated within intron 7 of *FKBP5* (Fig. 1a). These CpG sites (CpG542, 137,847 base pairs from the transcriptional start site and CpG543, 137,872 base pairs from the transcriptional start site) span experimentally validated glucocorticoid response elements^28^, and we and others have previously implicated methylation of these sites in metabolic disorders^13,14,28^. At baseline and following the 12-week intervention, CpG542/3 methylation was investigated in gluteal (GSAT) and abdominal subcutaneous adipose tissue (ASAT), as representatives of lower body and central obesity, respectively. Pyrosequencing analysis of GSAT samples in response to exercise training revealed that DNA methylation of CpG542 and CpG543 increased by 4% and 1.8%, respectively (Fig. 1b). However, these differences were only statistically significant for CpG542 (*p* = 0.019). In ASAT, methylation of both CpG542 and CpG543 increased by 9% in response to exercise training (Fig. 1b). In contrast, no significant differences in CpG542 or CpG543 methylation were observed in GSAT of control participants (Fig. 1c). While CpG542/3 methylation in ASAT tended to decrease post-intervention, these differences were not statistically significant (*p* > 0.05).

### FKBP5 methylation associations with obesity and metabolic parameters

**Figure 2:**
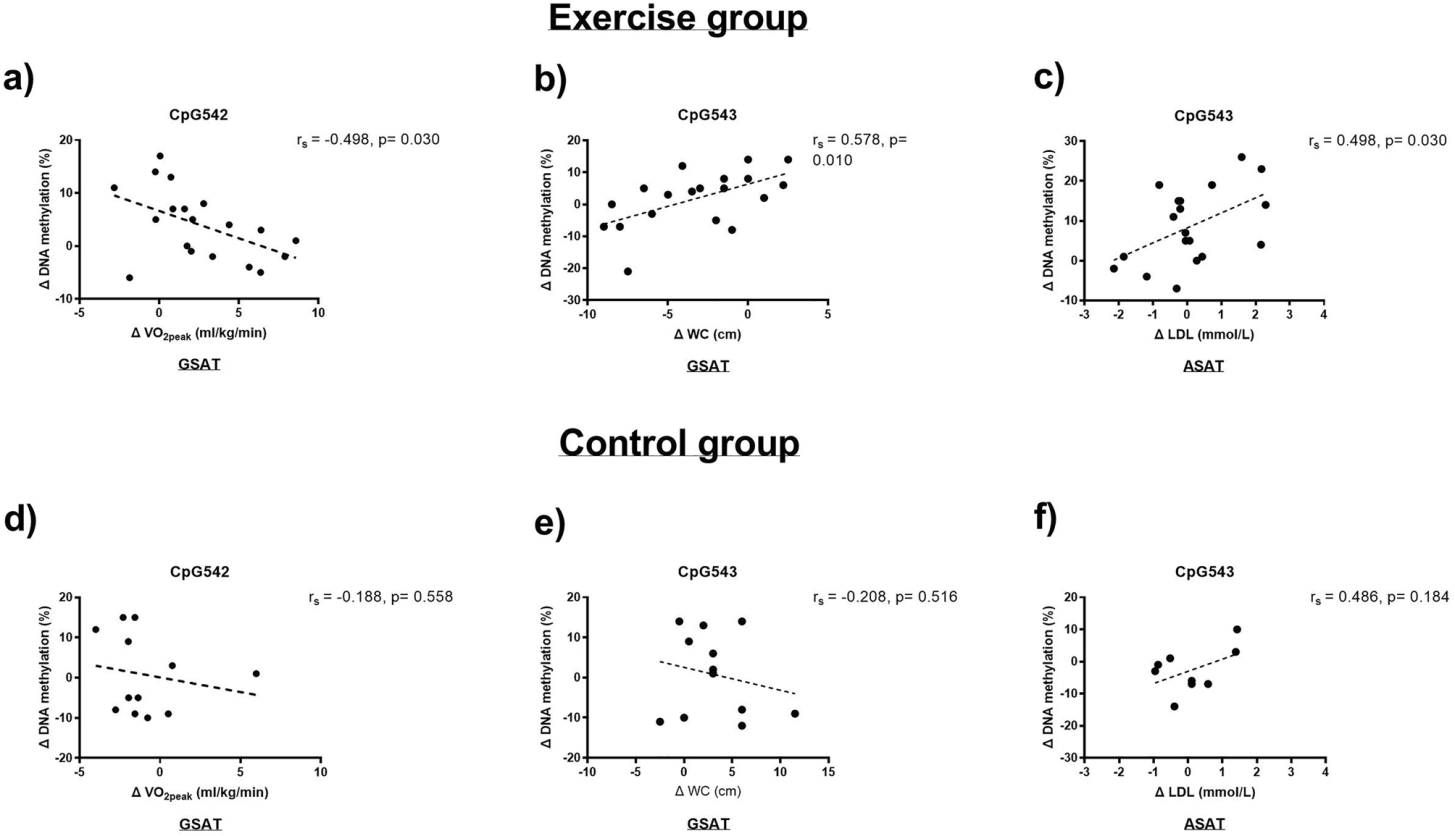
Spearman’s correlation analysis between changes in response to exercise training (Δ) in depot specific *FKBP5* methylation and Δ VO_2peak_ (**a**) and Δ waist circumference (WC) (**b**) and Δ low-density lipoprotein (LDL) levels (**c**) (n = 12–19 per group). Linear regression lines used for descriptive purposes only. GSAT, gluteal subcutaneous adipose tissue; ASAT, abdominal subcutaneous adipose tissue.

We next assessed associations between change in response to the intervention (Δ) in *FKBP5* CpG542 and CpG543 methylation and measures of cardiorespiratory fitness, insulin sensitivity and body composition. As shown in Fig. 2, Δ in GSAT CpG542 methylation negatively correlated with Δ cardiorespiratory fitness (VO_2peak_, r_s_ = −0.498, *p* = 0.03) and Δ CpG543 methylation positively associated with Δ WC (r_s_ = 0.578; *p* = 0.01) in response to exercise training (Fig. 2a, b). In ASAT, Δ CpG543 methylation positively associated with Δ low-density lipoprotein (LDL) cholesterol levels (r_s_ = 0.498, *p* = 0.03) in response to exercise training (Fig. 2c). Importantly, these associations were not significant in the control group (Fig. 2d-f). To control for socio-demographic and behavioural factors^29,30^, further linear regression models were adjusted for significant covariates (alcohol consumption and employment). Adjusted models showed that the associations between Δ CpG543 methylation and Δ WC (β = 1.26, *p* = 0.02) and Δ LDL (β = 3.51, *p* = 0.05) were maintained; however, the association between ΔCpG542 methylation and Δ cardiorespiratory fitness lost statistical significance (β = −0.01, *p* = 0.10).

### Effects of rs1360780 SNP on FKBP5 methylation and gene expression

The rs1360780 SNP, located in intron 2 of *FKBP5*, has previously been associated with metabolic risk^12,31,32^ and has been experimentally demonstrated to modulate glucocorticoid receptor-induced DNA methylation of *FKBP5* at multiple intronic CpG regions, including those in intron 7^28^. Based on this, we hypothesized that the rs1360780 SNP may influence *FKBP5* methylation and accordingly, gene expression levels.

**Figure 3:**
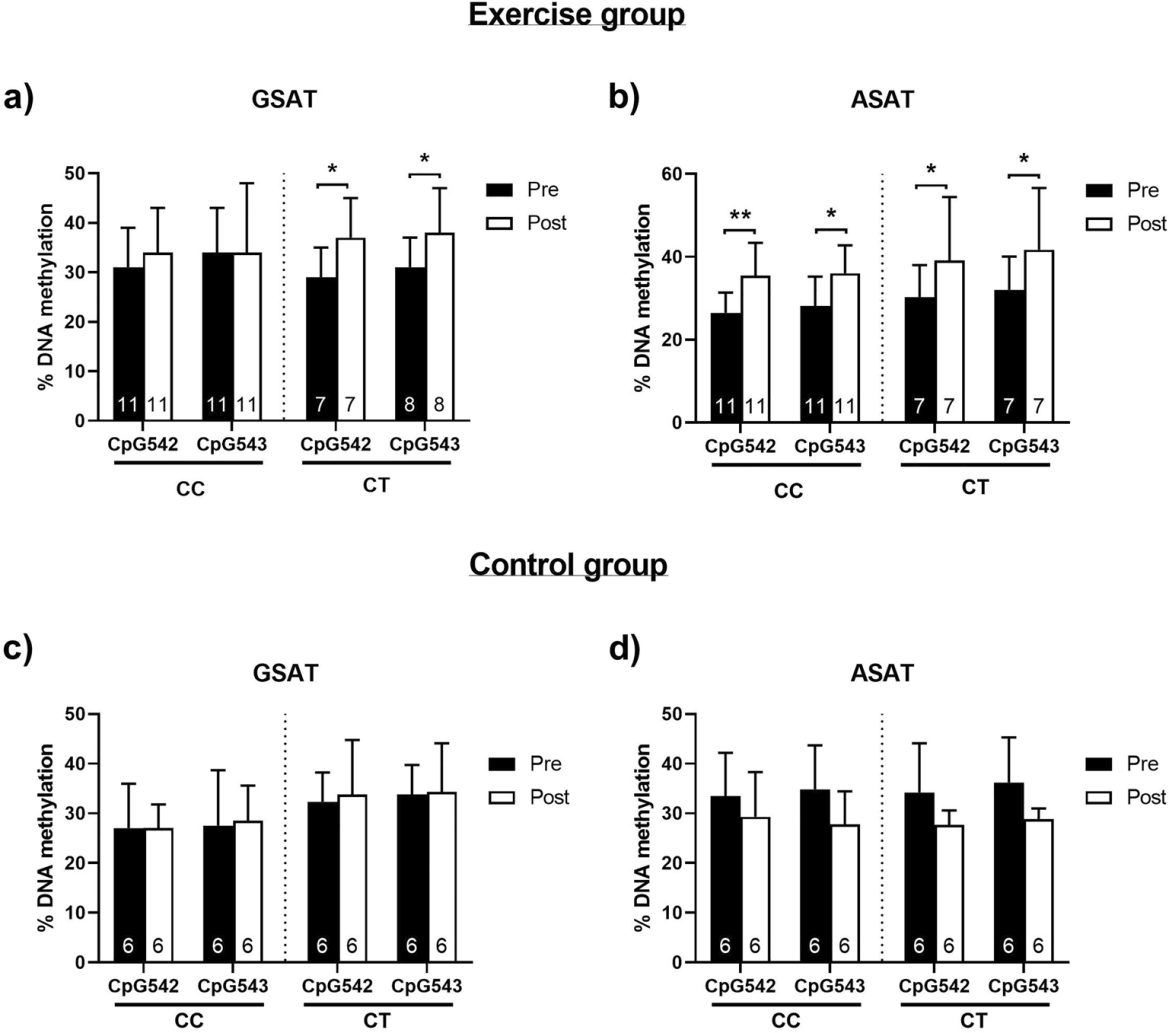
Genotype effect of rs1360780 on *FKBP5* CpG542 and CpG543 methylation in gluteal adipose tissue (GSAT) and abdominal adipose tissue (ASAT) in exercise participants (CC n = 11, CT n = 7) (**a, b**) and GSAT and ASAT in control participants (CC n = 6, CT n = 6) (**c, d**), pre- and post-intervention. Data represented as mean ± SD. *p < 0.05, **p < 0.01.

To explore this hypothesis, we genotyped all study participants for the presence of either the CC “protective allele” or the CT/TT “risk allele”. Using the combined sample of participants from the control and exercise groups (n = 30), we determined the genotype frequencies for the CC and CT alleles to be 0.57 (n = 17) and 0.43 (n = 13), respectively. For the exercise group, the genotype frequencies for the CC and CT alleles were 0.61 (n = 11) and 0.39 (n = 8), respectively. No participants were carriers of the rare homozygous TT genotype. We did not observe a significant deviation from the Hardy Weinberg equilibrium in the combined (*p* = 0.784) or exercise (*p* = 0.809) sample populations (data not shown). When participants from the exercise group were stratified based on the rs1360780 SNP genotype, those with the CT genotype presented with significantly higher GSAT CpG542 (*p* = 0.027) and CpG543 (*p* = 0.018) methylation in response to exercise training, whereas methylation of these CpG sites remained unchanged by exercise in individuals with the CC genotype (Fig. 3a). Conversely, in ASAT, both CC and CT genotypes displayed CpG542 and CpG543 hypermethylation after exercise training (Fig. 3b). No significant DNA methylation differences were observed in GSAT of either CC or CT control participants post intervention (Fig. 3c). While CpG542/3 methylation appeared to be lower in ASAT post exercise-training, these differences were not significant (Fig. 3d).

**Figure 4:**
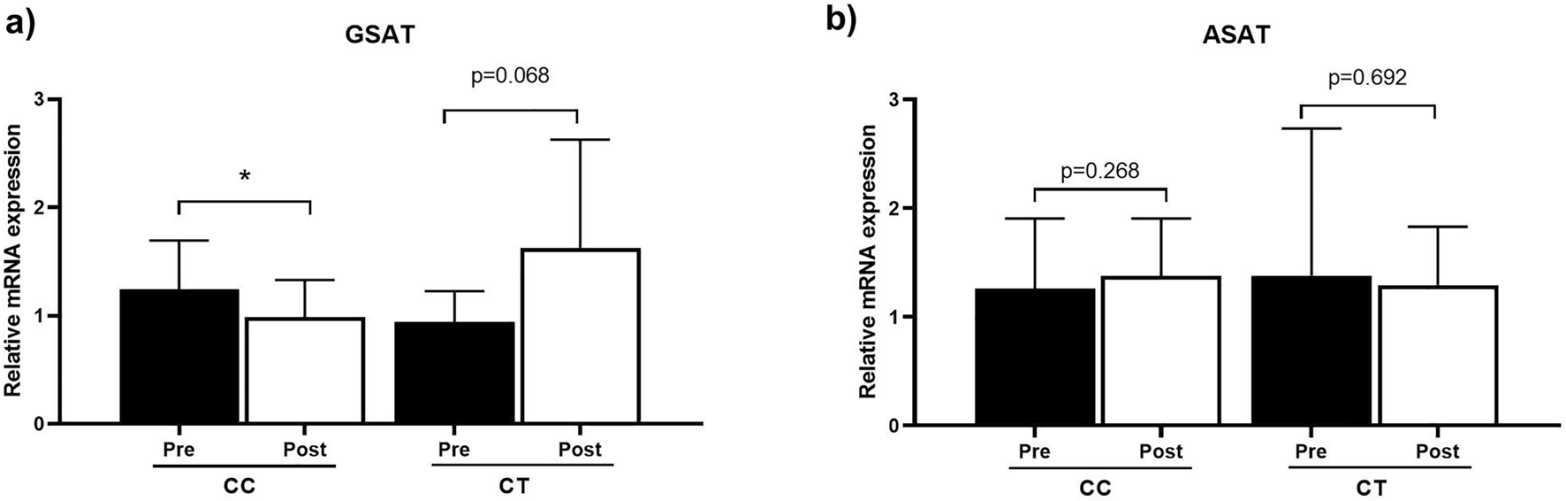
Relative mRNA expression of *FKBP5* in gluteal adipose tissue (GSAT) (**a**) and abdominal subcutaneous tissue (ASAT) (**b**) pre- and post-exercise training intervention and stratified by the rs1360780 genotype. Data represented as mean ± SD. *p < 0.05. (n = 19, CC n = 11, CT n = 7).

We next investigated *FKBP5* messenger ribonucleic acid (mRNA) levels in GSAT and ASAT in response to exercise training, which was compared between rs1360780 SNP genotype groups. As shown in Fig. 4a, a modest but significant decrease in *FKBP5* mRNA expression levels was observed in GSAT of individuals with the CC genotype after exercise training (*p* = 0.033), whilst individuals with the CT genotype displayed an apparent increase in *FKBP5* expression, although these changes were not significant (*p* = 0.068). A comparison of *FKBP5* mRNA expression in ASAT showed no significant differences between any of the groups in response to exercise training (Fig. 4b). In control participants, *FKBP5* expression levels in GSAT increased slightly in individuals with the CT genotype, although this increase was not significant (*p* = 0.866) (Fig. S1). In CC participants, *FKBP5* expression levels remained unchanged (Fig. S1).

### Effect of rs1360780 SNP on post-exercise training parameters

**Figure 5:**
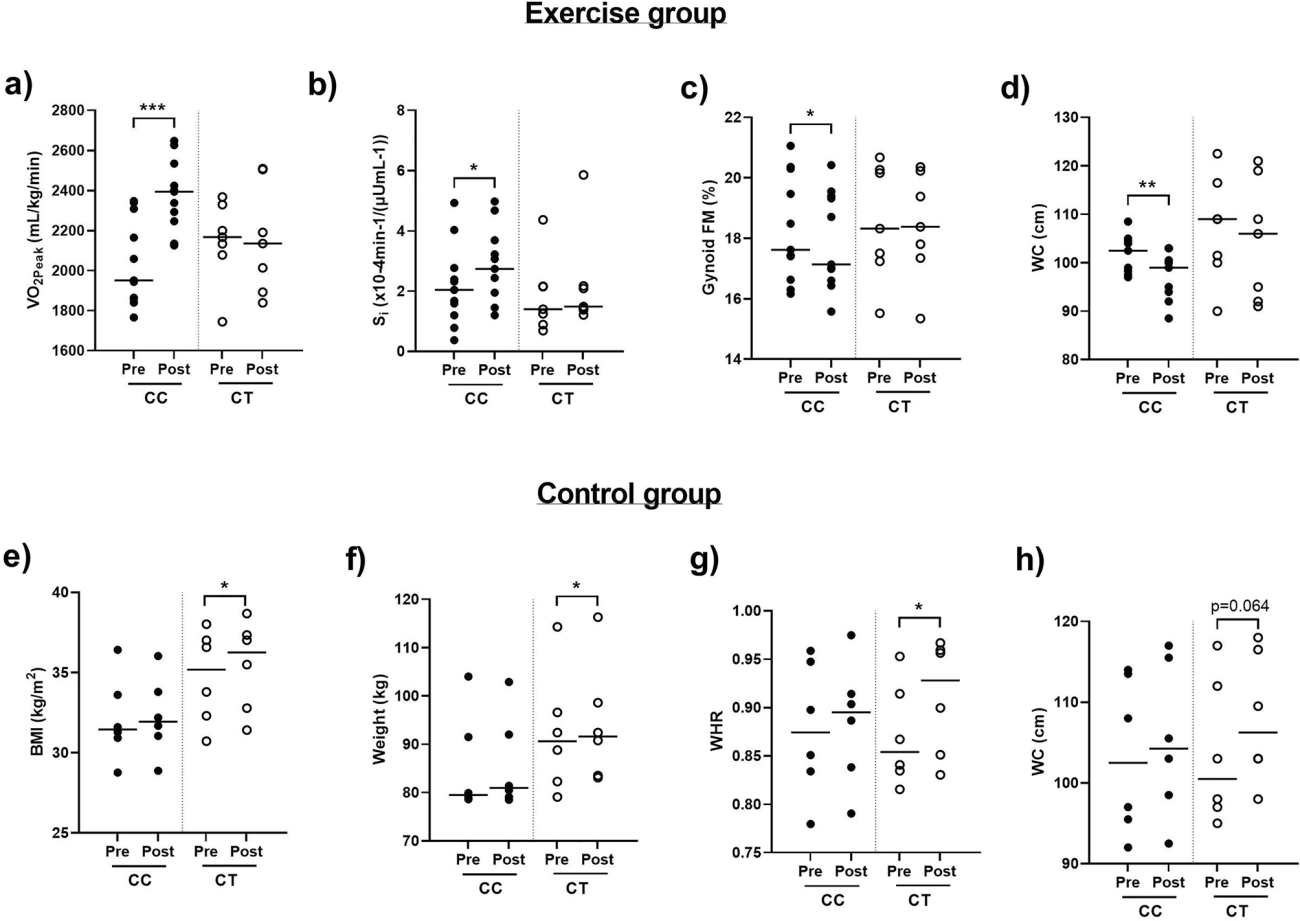
Individual changes in VO_2peak_ (**a**), waist circumference (WC) (**b**), gynoid fat mass % (FM) (**c**) and insulin sensitivity (S_I_) (**d**) in exercise group (n = 19, CC n = 11, CT n = 7) and body mass index (BMI) (**e**), weight (**f**), waist to hit ratio (WHR) (**g**) and WC (**h**) in control group (n = 12, CC n = 6, CT n = 6) pre- and post-intervention. Thick black line–median, shaded circles-CC genotype, empty circles–CT genotype. *p < 0.05, **p < 0.01, ***p < 0.001.

Based on the results in Figs. 3 and 4, and to better describe and quantify the metabolic differences between CC and CT carriers, we reinvestigated the participant characteristics pre- and post-intervention (Table 1) after stratifying for the rs1360780 SNP. Figure 5 shows that CC carriers displayed significant improvements in cardiorespiratory fitness (Fig. 5a) and insulin sensitivity (Fig. 5b), and reduced gynoid FM % (Fig. 5c) and WC (Fig. 5d) resulting from the exercise intervention, while these parameters were attenuated in individuals with the CT genotype. Furthermore, an investigation of the stratified control group revealed that the increased BMI, weight, waist to hip ratio and waist circumference observed in this group after the 12-week intervention period, as reported in Table 1, were only significant in participants with the CT genotype (Fig. 5e–h). These findings were not influenced by any of the sociodemographic or behavioural factors recorded in the study, as these parameters were not statistically different between the stratified groups (data not shown).

## Discussion

A growing list of studies have implicated HPA axis dysregulation in obesity aetiology^2,14,33,34^. We present novel pilot data that shows exercise training induced genotype-specific hypermethylation of *FKBP5*, an important regulator of the HPA axis, in GSAT and ASAT from South African women with obesity. Importantly, we showed that the improvements in cardiorespiratory fitness, insulin sensitivity and adiposity were attenuated in carriers of the *FKBP5* rs1360780 (C/T) SNP, although these findings need to be validated in a larger sample size.

Pyrosequencing analysis in this study revealed that 12 weeks of exercise training increased *FKBP5* CpG542 methylation in GSAT and both CpG542 and CpG543 in ASAT. Interestingly, previously studies performed in our laboratory demonstrated that *FKBP5* is hypermethylated in GSAT and ASAT of obese compared to normal-weight South African women^13^. While these findings seem contradictory to those reported here, it is important to note that the previous study was a cross-sectional comparison between *FKBP5* methylation in normal weight compared to obese women, who did not undergo any intervention pre- and post- methylation analysis. In the current study, CpG542 methylation in GSAT associated with decreased cardiorespiratory fitness, an association which has, to the best of our knowledge, not been previously described. Moreover, depot specific *FKBP5* CpG543 methylation was associated with increased WC (GSAT) and LDL levels (ASAT), which agrees with findings from a previous study by Ortiz and colleagues^14^, which showed that hypermethylation of *FKBP5* within intron 2 in peripheral blood positively associated with WC and LDL in type 2 diabetic individuals. In line with this, we also previously reported a positive relationship between GSAT *FKBP5* CpG542 and CpG543 methylation and WC in obese compared to normal-weight South African women^13^.

Studies on the *FKBP5* functional rs1360780 SNP (C/T) have shown that the presence of the T allele may influence susceptibility to obesity and metabolic disease^12^. Indeed, carriers of the CT “risk allele” were shown to display higher insulin resistance, fasting insulin, circulating triglycerides and HOMA-IR values compared to wildtype CC carriers^12^. Furthermore, studies focusing on the role of the rs1360780 SNP in weight loss outcomes have shown that carriers of the T allele lose less weight in comparison to CC allele carriers following bariatric surgery^31,35^. In the current study, participants with the CC genotype showed significant improvements in cardiorespiratory fitness, insulin sensitivity and adiposity after exercise training, whilst these parameters were not significantly altered by exercise training in individuals with the CT genotype. Similarly, while the control group displayed significantly increased weight, BMI and waist circumference after the 12-week intervention period, stratification of these individuals according to the rs1360780 SNP revealed that these parameters were only significantly increased in CT carriers. Taken together, these finding agree with literature stating that CC carriers are more responsive to interventions associated with improvements in metabolic health compared to CT carriers, although we acknowledge that this requires validation in additional larger datasets^12,31,35^. Furthermore, although CT carriers did not display improvements in response to exercise training in the current study, we acknowledge that exercise may still be an appropriate intervention for the prevention of weight gain in these individuals.

Another important finding of the current study is that *FKBP5* transcript levels were significantly downregulated by exercise in GSAT of CC allele carriers, while in contrast, CT carriers tended to display higher *FKBP5* levels post-exercise intervention. We speculate that the gene expression changes for CT carriers may have not reached statistical significance due to the smaller sample size available for this genotype. Nonetheless, these findings agree with earlier reports showing that the rs1360780 (C/T) SNP is associated with higher *FKBP5* induction by glucocorticoids^36^. Indeed, Klengel et al.^36^. showed that this is due to the T allele resulting in the formation of a putative TATA box, which interacts with the *FKBP5* transcription start site through long-range chromatin interactions, thus enhancing *FKBP5* expression^36^. The induction of *FKBP5* in CT carriers observed here also agrees with previous preclinical studies showing that loss of FKBP5 in mice fed a high fat diet improves their metabolic health^8^, whilst higher FKBP5 levels in humans is associated with obesity and negative metabolic outcomes^11^. Importantly, while we did not observe any significant differences in baseline *FKBP5* mRNA levels between CC and CT carriers, we note that other studies have similarly shown that FKBP5-mediated biological effects are evident only after the introduction of an environmental challenge, such as the exposure to a high fat diet^4,8^, bariatric surgery^31,35^ or psychological trauma and stress^6,37,38^. Interestingly, we did not observe any significant differences in *FKBP5* gene expression in ASAT after exercise training. We suspect that this may be due to intrinsic transcriptome differences between GSAT and ASAT, as demonstrated in a previous study by Nono Nankam et al.^22^ which assessed these tissues from the same study population^22^.

**Figure 6:**
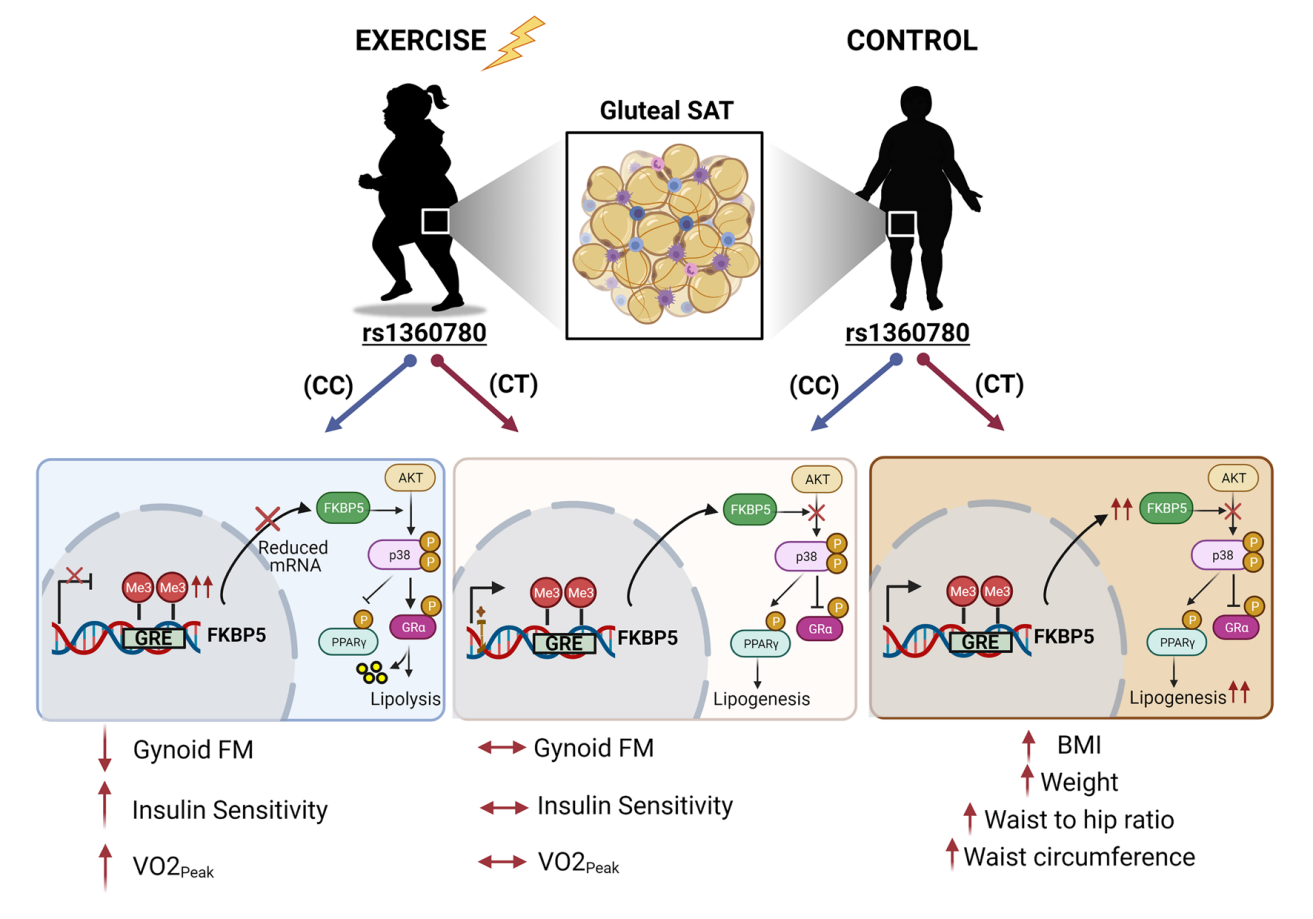
Schematic proposed model of study findings showing how exercise intervention can lead to intronic hypermethylation and downregulation of *FKBP5* expression in individuals harbouring “protective” rs1360780 CC genotype. Reduced FKBP5 in turn may lead to re-activation of the Akt-p38 MAPK pathway, favouring GRα-induced lipolysis of gynoid fat mass (FM) and consequently improving insulin sensitivity and cardiorespiratory fitness. In contrast, individuals carrying the CT “risk” allele undergo sustained FKBP5 induction, causing a suppression of Akt-p38 MAPK signalling and promotion of lipogenesis through PPARγ activation. These molecular events lead to an attenuated response to exercise intervention. In the control group, the presence of the CT allele may result in an even greater suppression of the Akt-p38 MAPK pathway and adipogenic hyperactivity, resulting in increased weight gain over time compared to CC carriers. Diagram created in Bio-Render (https://biorender.com/).

The biological significance of *FKBP5* gene expression changes in GSAT in response to exercise training was not experimentally explored in our study due to limited sample availability. However, it has been previously shown that *FKBP5* knockdown in mature 3T3-L1 adipocytes reduced lipid accumulation and expression of adipogenic genes, and *FKBP5* knockout in mice fed a high fat diet prevented white adipose tissue accumulation compared to wildtype controls^7,9^. This was mechanistically shown in the latter study to occur through FKBP5-mediated inhibition of the Akt-p38 MAPK pathway, which resulted in a reduction in the pro-lipolytic activity of GRα and promotion of lipogenesis by PPARγ, both of which are direct targets and reciprocally regulated by p38 MAPK phosphorylation^7,9^. According to this model, we propose that the reduction in *FKBP5* in GSAT of CC participants in response to exercise training may have resulted in GRα activation via loss of Akt-p38 MAPK signaling, thus favouring and resulting in lipolysis of gynoid fat^39^. This in turn, may have contributed to the corresponding improvement in cardiorespiratory fitness and insulin sensitivity—a relationship previously demonstrated in this study population^39^. On the other hand, participants harbouring the rs1360780 (C/T) SNP retained elevated *FKBP5* levels, and consequently, continued suppression of Akt-p38 MAPK signalling and sustained PPARγ-mediated adipogenic and lipogenic activity (Fig. 6). In the control group, suppression of the Akt-p38 MAPK pathway, and consequent adipogenic hyperactivity, may have been exacerbated in CT carriers, who continued their usual behaviours over the 12-week intervention period, and whose *FKBP5* levels in GSAT appeared to increase compared to control participants harbouring the CC allele. We speculate that in this scenario, the rs1360780 (C/T) SNP may interact with an obesogenic environment to cause a genetic vulnerability to metabolic dysregulation, resulting in increased weight gain over time compared to CC carriers, as observed in this study (Fig. 6).

Our study has several strengths. To the best of our knowledge, this is the first study investigating *FKBP5* methylation, the rs1360780 SNP and gene expression changes in response to an exercise intervention in an exclusively African population of women with obesity. These findings are important since most available DNA methylation and transcriptome datasets have originated from studies performed in high-income countries and in European populations and may thus not be extrapolated to other nations with different ethnic and economic backgrounds. Furthermore, the current study investigated the impact of exercise on *FKBP5* methylation in multiple adipose tissue depots, the primary affected organ during obesity development, compared to other studies which used peripheral blood^14,31,35^. As the participants in this study were advised to maintain their diet, no differences in energy consumption were found pre- and post-intervention, thus allowing for the exclusion of dietary influence on *FKBP5* methylation. An additional strength of this study is the inclusion of both gluteal and abdominal subcutaneous adipose tissues in our analyses, thus enabling direct comparison between these depots and their associations with body composition and metabolic parameters.

Our study also has several limitations that need to be considered. Since the collection of adipose tissue biopsies is an invasive procedure, the study sample size is small^27^. Furthermore, due to a higher dropout rate in the control group, the sample sizes for the exercise and control groups were not equal and this is thus a limitation of the study^27^. Retrospective analysis of the statistical power for outcomes described in this pilot study, from which our main conclusions were drawn, showed that the power of these tests ranged between 60–90%. We therefore acknowledge that this study is underpowered to accurately assess the effects of *FKBP5* methylation and the rs1360780 SNP on exercise outcomes. As a result, this study is a hypothesis generating pilot analysis, and future studies should investigate whether the associations reported here can be confirmed in additional, larger datasets. This study also only investigated an exercise intervention in South African women with obesity, and thus we cannot extrapolate our findings to men, or other ethnic populations. Finally, due to limited tissue availability, we could not measure FKBP5 protein levels in GSAT and ASAT, and therefore, cannot assess whether the changes in DNA methylation and gene expression were associated with functional FKBP5 protein levels.

In conclusion, we showed that a 12-week exercise intervention induced *FKBP5* hypermethylation in a cohort of women with obesity. We found a significant relationship between *FKBP5* methylation and measures of central obesity (WC), cardiorespiratory fitness (VO_2peak_) and hyperlipidaemia (LDL levels), which are important determinants of metabolic health. *FKBP5* methylation, together with the effects of the rs1360780 SNP on the aforementioned parameters highlights the potential of dysregulated (epi)genetic processes to influence metabolic risk. These findings provide a basis for future studies in larger cohorts, which should assess whether *FKBP5* methylation and/or genetic variants such as the rs1360780 SNP could have a significant impact on responsiveness to exercise interventions.

## Methods

### Study design

This is a pilot analyses of a larger randomized control trial (RCT), for which details of the study design, participant selection and methods have been described previously^27^. The study was approved by the UCT Human Research Ethics Committee (HREC REF: 054/2015) and was performed in accordance with principles of the Declaration of Helsinki (1964, amended in 2013). The trial was registered in the Pan African Clinical Trial Registry (Clinical trial number: PACTR201711002789113, registered on 21/11/2017). Written, informed consent was obtained from all participants prior to screening and participation. Sedentary, South African women with obesity were recruited through advertisements and selected based on the following inclusion criteria: (1) Black, South African women (based on parental Xhosa ancestry) between the ages of 20–35 years; (2) classified with obesity (BMI 30–40 kg/m^2^); (3) weight stable (no change in weight more than 5 kg/no change in clothing size over 6 months prior to selection); (4) sedentary (within the last 12 months had not participated in exercise training (more than 1 session lasting more than 20 min per week); (5) on injectable contraceptive (minimum 2 months; depot medroxyprogesterone acetate 400 mg); (6) no known metabolic/inflammatory disease; (7) no hypertension (≥ 140/90 mmHg), diabetes (random plasma glucose concentration > 11.1 mmol/l or HbA1C > 6.5%); (8) not currently on any medications; (9) non-smokers; (10) not currently pregnant/lactating; (11) no medical problems preventing participation in training; and (12) no surgical procedures 6 months prior to study. A total of 118 women were assessed for eligibility, of which 45 women partook in the study. Based on the availability of samples, 31 of these participants were included in our study, of which 12 and 19 participated in the control and exercise groups, respectively.

### 12-week intervention

The study participants were randomly divided into a control or experimental (exercise intervention) group. The control group was instructed to maintain their normal daily activities while the intervention group underwent 12 weeks of supervised aerobic/resistance training at moderate/vigorous intensity (60–70% maximal heart rate) for 40–60 min four times a week. Intensity of training was maintained throughout the study by adjusting exercise activity, which was monitored using heart rate monitors (Polar A300, Kempele, Finland). All participants were instructed to maintain their usual dietary habits throughout the duration of the study, which was assessed monthly as described previously^39^. All participants underwent pre- and post-testing; where anthropometric, metabolic and biochemical data were obtained, and adipose samples were collected, as previously described and detailed below^27^.

### Pre and post intervention testing

#### Cardiorespiratory fitness

Peak oxygen consumption (VO_2peak_), a measure of cardiorespiratory fitness, was assessed as described previously^27^. Briefly, a walking treadmill-based (C, Quasar LE500CE, HP Cosmos, Nussdorf-Traunstein, Germany) graded exercise test was utilized, which involved an increase in treadmill gradient, followed by alternate increases in speed and gradient until volitional exhaustion was achieved. Pulmonary gas exchange was quantified by measuring the concentrations of O2 and CO2 and ventilation, from which VO2 consumption was calculated using a metabolic gas analysis system (CPET, Cosmed, Rome Italy).

#### Body composition

Basic anthropometric measurements (weight, height, waist and hip circumference) were measured. Whole body composition, including fat mass as well as regional body (gynoid and android) fat mass distribution, were assessed using dual-energy-x-ray absorptiometry (DXA) (Discovery-W, Software version 12.7.3.7; Hologic Inc., Medford, USA). MRI was used to determine visceral adipose tissue (VAT) and abdominal adipose tissue (SAT) using a 3 Tesla whole-body human MRI scanner (MAGNETOM Skyra; Siemens Medical Solutions). Region of interests (ROIs) were manually drawn using HOROS V 1.1.7 and volumes were determined by calculating the sum of the VAT and SAT areas from five images in a 15 cm region from the level of L1-5 and then multiplied by 3^40^.

#### Frequently sampled intravenous glucose tolerance test

Following an overnight fast, blood samples were collected for the quantification of fasting glucose, insulin, triglycerides, total cholesterol, LDL and HDL. Thereafter, insulin sensitivity was calculated using an insulin-modified frequently sampled intravenous glucose tolerance test, as previously described^41^, from which the insulin sensitivity index (S_I_) was determined using Bergman’s minimal model of glucose kinetics^42^.

#### Biochemical analysis

Plasma glucose concentrations and serum lipids were measured using colorimetric assays (Randox, Gauteng, South Africa), and serum insulin was determined using immunochemiluminometric assays (IMMULITE 1000 immunoassay system, Siemens Healthcare, Midrand, South Africa). Plasma glucose and serum insulin concentrations were used to estimate insulin resistance, based on the homeostasis model of insulin resistance (HOMA-IR)^43^.

#### Adipose tissue biopsies

SAT biopsies (2–3 cm^3^) were obtained during both testing periods via mini liposuction following a 4–6 h fast^44^. Abdominal subcutaneous adipose tissue (ASAT) was obtained from the area directly above the umbilicus, whereas gluteal subcutaneous adipose tissue (GSAT) was obtained from the upper, right, outer quadrant of the gluteal region^27^.

#### DNA extraction

Genomic DNA from approximately 100 mg of GSAT and ASAT was isolated using the Qiagen DNeasy Blood & Tissue Kit (Qiagen, Valencia, USA) as per the manufacturer’s instructions. DNA purity and quantity were assessed using the Nanodrop Spectrophotometer (Nanodrop™ One, Thermo Fisher Scientific, Waltham, USA) and Qubit fluorometer (Life Technologies, CA, USA), respectively.

#### Bisulfite pyrosequencing

Bisulfite conversion and PCR amplification were carried out using the EpiTect Bisulfite Conversion kit (Qiagen, Hilden, Germany) and PyroMark PCR kit (Qiagen, Hilden, Germany), respectively, according to the manufacturer’s instructions (Qiagen, Hilden, Germany). FKBP5 (assay ID is ADS3828) primers were designed and purchased from EpigenDX (Worcester, MA, USA). The sequence of the primers was not disclosed by the company. The PCR reaction was carried out in the Veriti 96-well Thermal Cycler (ThermoFisher, Waltham, MA, USA) and the quality of amplicons was assessed by agarose gel electrophoresis. Amplicons were visualised using the ChemiDoc Imaging System (BioRad, Hercules, CA, USA). Pyrosequencing was conducted on the PyroMark Q96 using the PyroMark Gold Kit (Qiagen, Valencia, CA, USA), as previously described^13^. Each pyrosequencing run contained bisulfite conversion and PCR no template negative controls. Bisulfite conversion controls were also incorporated within each assay sequence to assess conversion efficiency. Assays were repeated if any of the inbuilt quality control measures failed. The nomenclature of the two FKBP5 CpGs are based on their sequencing identities from EpigenDX (Worcester, MA, USA).

#### FKBP5 genotyping

The FKBP5 rs1360780 (C/T) single nucleotide polymorphisms (SNPs) were genotyped as previously described^13^. Briefly, quantitative real-time PCR (qRT-PCR) was performed using Taqman genotyping assays (Applied Biosystems, Massachusetts, USA) on the QuantStudio™ 7 Flex Real-Time PCR System; with each reaction containing 9.4 ng of DNA, 5 µl of Taqman Master Mix and 0.25 µl of 40X TaqMan SNP genotyping assay in a total volume of 10 µl (Applied Biosystems, Massachusetts, USA). The PCR conditions were as follows: 10 min at 95 °C (initial denaturation/enzyme activation), 15 s at 95 °C (denaturation) and 60 s at 60 °C (annealing/extension) for 40 cycles. Eight randomly selected samples, genotyped in duplicate, were included as quality controls; with all plates including positive and negative controls.

#### RNA extraction, reverse transcription and quantitative real-time PCR

Total RNA was extracted from adipose tissue biopsies using the RNeasy/miRNeasy mini Kit (Qiagen, CA, United States) as previous described^13^, and assessed for quantity and purity using the NanoDrop ND-1000 Spectrophotometer (Agilent Technologies, Boeblingen, Germany). Complementary DNA (cDNA) was synthesized using the High Capacity cDNA Reverse Transcription Kit (Applied Biosystems, CA, USA). Gene expression was quantified using TaqMan Universal PCR Master Mix and TaqMan gene expression assays (Applied Biosystems, CA, USA), according to the manufacturer’s instructions. Relative gene expression levels were calculated using the standard curve method, and FKBP5 expression levels were normalised to the RPLP0 endogenous control (identified as the best normalization gene using Normfinder)^45^.

### Statistical analysis

This is a pilot analyses of a larger RCT, which has previously described the power analysis and sample size determination based on the primary outcomes relating to insulin sensitivity^27^. An adjustment for multiple testing was not considered due to the small sample size of this pilot analyses. Statistical analysis was conducted using GraphPad Prism (v.8.4.2) and STATA version 14.0 (StataCorp, College station, TX, USA). Data were assessed for normality using the Shapiro–Wilk test (*p* < 0.05) and are presented as the mean and standard deviation (SD) or the median and interquartile range (25th and 75th percentile) if normally or not normally distributed, respectively. Mixed-model analyses with main effects of time (pre- and post-intervention) and group (exercise and control) and interaction effects (group x time) were used to assess anthropometric, biochemical and metabolic parameters. DNA methylation and gene expression of *FKBP5* were tested for significant changes pre- and post- exercise intervention using Paired Student T-Tests (normal distribution) or the non-parametric Wilcoxon Signed-Rank Tests (non-normal distribution), as appropriate. The correlation between the change (post-exercise training from baseline) in *FKBP5* DNA methylation and metabolic parameters (cardiorespiratory fitness, body composition, glucose regulation and lipid profile) were determined using Pearson’s (normal distribution) or Spearman’s (non-normal distribution) correlation tests. Linear regression was used to explore any significant effects of social and behavioural factors on DNA methylation. Employment and alcohol consumption were determined to be significant covariates and were thus included in the adjusted model. Smoking was not considered as a covariate as no participants were current or previous smokers. Statistical significance was accepted as *P* < 0.05.

### Ethics approval and consent to participate

This study was approved by the Research Ethics Committee of the Faculty of Health Sciences of the University of Cape Town and written informed consent to participate in the study was obtained from all subjects prior to participation in the study.

## Supplementary Information

Supplementary Information.

## Data Availability

The datasets generated and/or analysed in the current study are not publicly available as the study has only recently been completed. Data will be made publicly available after a two-year period; however, it can be made available from the corresponding author on reasonable request.
