# Supplementary material for: A pilot investigation of genetic and epigenetic variation of FKBP5 and response to exercise intervention in African women with obesity
Source: Sci Rep. 2022 Jul 11;12:11771. doi: 10.1038/s41598-022-15678-6 (PMC9273786; doi:10.1038/s41598-022-15678-6)
Supplement: Supplementary file 1 — Supplementary Information. [file 41598_2022_15678_MOESM1_ESM.docx]

**Supplementary figures**

**
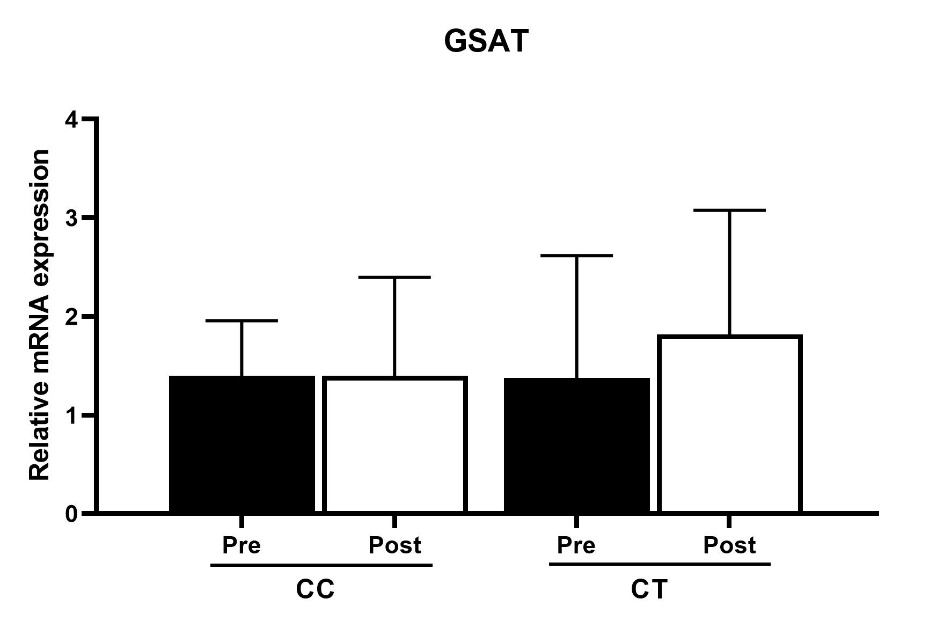
**

**Figure S1.** Relative mRNA expression of *FKBP5* in gluteal adipose tissue (GSAT) of control participants pre and post exercise intervention and stratified by the rs1360780 genotype. Data represented as mean ± SD (n=6 per group).

**Supplementary Tables**

**Table S1.** Correlation between the change (Δ) in *FKBP5* methylation levels and Δ cardiometabolic parameters in exercise group.

|  | GSAT | | | | | | ASAT | | | | | |
| --- | --- | --- | --- | --- | --- | --- | --- | --- | --- | --- | --- | --- |
|  | **CpG542** | | | **CpG543** | | | **CpG542** | | | **CpG543** | |  |
|  | **r** | **P** | **P*** | **r** | **P** | **P*** | **r** | **P** | **P*** | **r** | **P** | **P*** |
| Δ VO_2Peak_ (ml/kg) | **-0.498** | **0.030** | 0.100 | -0.191 | 0.433 | 0.806 | -0.188 | 0.273 | 0.485 | -0.311 | 0.195 | 0.393 |
| Body Composition and Fat Distribution | | | | | | | | | | | | |
| Δ BMI (kg/m^2^) | 0.405 | 0.085 | 0.203 | 0.344 | 0.149 | 0.322 | -0.196- | 0.421 | 0.231 | -0.115 | 0.639 | 0.393 |
| Δ Weight (kg) | 0.408 | 0.083 | 0.208 | 0.355 | 0.136 | 0.323 | -0.205 | 0.399 | 0.221 | -0.130 | 0.597 | 0.361 |
| Δ WHR | 0.165 | 0.499 | 0.711 | 0.229 | 0.347 | 0.502 | -0.137 | 0.576 | 0.447 | 0.013 | 0.957 | 0.877 |
| ΔWC (cm) | 0.436 | 0.062 | 0.187 | **0.578** | **0.010** | 0.024 | -0.174 | 0.475 | 0.200 | -0.130 | 0.597 | 0.283 |
| Δ FM (%) | 0.399 | 0.091 | 0.362 | 0.329 | 0.170 | 0.628 | 0.446 | 0.056 | 0.143 | 0.389 | 0.100 | 0.247 |
| Δ Android FM (%) | 0.039 | 0.873 | 0.418 | -0.212 | 0.383 | 0.866 | -0.126 | 0.375 | 0.408 | -0.085 | 0.729 | 0.870 |
| Δ Gynoid FM (%) | 0.179 | 0.464 | 0.409 | 0.249 | 0.304 | 0.228 | 0.343 | 0.150 | 0.135 | 0.280 | 0.245 | 0.230 |
| Glucose Regulation | | | | | | | | | | | | |
| Δ HbA1C (%) | 0.252 | 0.298 | 0.445 | 0.020 | 0.936 | 0.783 | 0.292 | 0.225 | 0.391 | 0.407 | 0.083 | 0.150 |
| Δ HOMA-IR | -0.073 | 0.768 | 0.750 | -0.069 | 0.778 | 0.602 | -0.117 | 0.634 | 0.809 | -0.206 | 0.397 | 0.572 |
| Δ Fasting Glucose (mmol/L) | 0.163 | 0.506 | 0.812 | 0.259 | 0.285 | 0.470 | 0.247 | 0.309 | 0.479 | 0.087 | 0.723 | 0.963 |
| Δ Fasting Insulin (μU/ml) | -0.106 | 0.665 | 0.709 | -0.109 | 0.656 | 0.556 | -0.298 | 0.215 | 0.343 | -0.334 | 0.163 | 0.298 |
| Δ Si (x10^-4^min^-1^/μUmL^-1^) | 0.242 | 0.318 | 0.086 | 0.024 | 0.924 | 0.418 | -0.022 | 0.928 | 0.898 | 0.022 | 0.930 | 0.729 |
| Lipid profile | | | | | | | | | | | | |
| Δ LDL (mmol/L) | 0.147 | 0.547 | 0.773 | 0.052 | 0.834 | 0.827 | 0.270 | 0.264 | 0.349 | **0.498** | **0.030** | **0.050** |
| Δ HDL (mmol/L) | 0.089 | 0.718 | 0.555 | 0.093 | 0.706 | 0.441 | 0.234 | 0.336 | 0.338 | 0.174 | 0.477 | 0.453 |
| Δ Triglycerides (mmol/L) | -0.012 | 0.961 | 0.658 | -0.340 | 0.155 | 0.257 | 0.048 | 0.845 | 0.620 | 0.241 | 0.321 | 0.184 |

Data expressed as the Pearson’s r-coefficient/Spearman’s rho coefficient and p-value. *P-value adjusted for alcohol consumption and employment

Abbreviations: BMI, body mass index; C-peptide; FM, fat mass; HbA1C, haemoglobin A1C ; HDL, high-density lipoprotein; HbA1C, haemoglobin A1C ; HOMA-IR, homeostatic model assessment of insulin resistance; LDL, low-density lipoprotein; Si, insulin sensitivity; WHR, waist-to-hip ratio; WC, waist circumference.

**Table S2.** Correlation between the change (Δ) in *FKBP5* methylation levels and Δ cardiometabolic parameters in control group.

|  | GSAT | | | | | | ASAT | | | | | |
| --- | --- | --- | --- | --- | --- | --- | --- | --- | --- | --- | --- | --- |
|  | **CpG542** | | | **CpG543** | | | **CpG542** | | | **CpG543** | |  |
|  | **r** | **P** | **P*** | **r** | **P** | **P*** | **r** | **P** | **P*** | **r** | **P** | **P*** |
| Δ VO_2Peak_ (ml/kg) | -0.188 | 0.558 | 0.540 | -0.007 | 0.538 | 0.669 | -0.005 | 0.710 | 0.794 | -0.008 | 0.538 | 0.669 |
| Body Composition and Fat Distribution | | | | | | | | | | | | |
| Δ BMI (kg/m^2^) | -4.793 | 0.388 | 0.354 | -6.692 | 0.249 | 0.178 | -5.162 | 0.446 | 0.446 | -6.693 | 0.249 | 0.178 |
| Δ Weight (kg) | -1.602 | 0.473 | 0.426 | -2.372 | 0.311 | 0.223 | -1.977 | 0.467 | 0.461 | -2.373 | 0.311 | 0.223 |
| Δ WHR | -28.316 | 0.738 | 0.711 | -21.292 | 0.813 | 0.600 | -60.546 | 0.553 | 0.491 | -21.292 | 0.813 | 0.600 |
| ΔWC (cm) | -0.719 | 0.381 | 0.389 | -0.208 | 0.516 | 0.396 | -0.627 | 0.534 | 0.515 | -0.169 | 0.847 | 0.751 |
| Δ FM (%) | -0.290 | 0.126 | 0.278 | -0.261 | 0.203 | 0.511 | -0.089 | 0.715 | 0.931 | -0.111 | 0.596 | 0.770 |
| Δ Android FM (%) | -1.878 | 0.116 | 0.255 | -1.806 | 0.159 | 0.411 | -0.742 | 0.629 | 0.820 | -0.851 | 0.519 | 0.678 |
| Δ Gynoid FM (%) | -0.887 | 0.109 | 0.244 | -0.822 | 0.169 | 0.443 | -0.290 | 0.685 | 0.900 | -0.342 | 0.578 | 0.746 |
| Glucose Regulation | | | | | | | | | | | | |
| Δ HbA1C (%) | -20.228 | 0.020 | 0.036 | -17.862 | 0.065 | 0.082 | 9.878 | 0.406 | 0.302 | 6.763 | 0.513 | 0.457 |
| Δ HOMA-IR | -1.405 | 0.602 | 0.545 | -1.639 | 0.566 | 0.576 | 2.455 | 0.450 | 0.472 | 2.260 | 0.419 | 0.395 |
| Δ Fasting Glucose (mmol/L) | -1.959 | 0.566 | 0.437 | -2.077 | 0.566 | 0.376 | 8.847 | 0.014 | 0.032 | 5.575 | 0.097 | 0.155 |
| Δ Fasting Insulin (μU/ml) | -0.398 | 0.562 | 0.520 | -0.464 | 0.524 | 0.594 | -0.687 | 0.407 | 0.419 | -0.321 | 0.658 | 0.751 |
| Δ Si (x10^-4^min^-1^/μUmL^-1^) | -2.325 | 0.176 | 0.238 | -2.606 | 0.150 | 0.128 | -0.239 | 0.913 | 0.975 | -0.770 | 0.682 | 0.639 |
| Lipid profile | | | | | | | | | | | | |
| Δ LDL (mmol/L) | 3.243 | 0.255 | 0.167 | 3.014 | 0.322 | 0.170 | 5.042 | 0.136 | 0.118 | 5.786 | 0.184 | 0.290 |
| Δ HDL (mmol/L) | -5.274 | 0.661 | 0.765 | -3.432 | 0.788 | 0.843 | 22.080 | 0.108 | 0.118 | 22.633 | 0.048 | 0.063 |
| Δ Triglycerides (mmol/L) | -6.455 | 0.506 | 0.663 | -2.924 | 0.779 | 0.960 | 10.107 | 0.388 | 0.346 | 4.399 | 0.668 | 0.581 |

Data expressed as the Pearson’s r-coefficient/Spearman’s rho coefficient and p-value. *P-value adjusted for alcohol consumption and employment

Abbreviations: BMI, body mass index; C-peptide; FM, fat mass; HbA1C, haemoglobin A1C ; HDL, high-density lipoprotein; HbA1C, haemoglobin A1C ; HOMA-IR, homeostatic model assessment of insulin resistance; LDL, low-density lipoprotein; Si, insulin sensitivity; WHR, waist-to-hip ratio; WC, waist circumference
